# Therapeutic Challenges in Managing Triple-Negative Breast Cancer in a Patient With Central Core Disease

**DOI:** 10.7759/cureus.94034

**Published:** 2025-10-07

**Authors:** Fatima Ibrahim, Eeman Noor, Asfandyar Khalil, Usman Sehbai, Aasim Sehbai

**Affiliations:** 1 Medicine, CMH Lahore Medical College and Institute of Dentistry, Lahore, PAK; 2 Medicine, Swat Medical College, Swat, PAK; 3 Internal Medicine, Health Department Khyber Pakhtunkhwa, Peshawar, PAK; 4 Oncology, Alabama Cancer Care Center, Anniston, USA; 5 Hematology and Oncology, Alabama Cancer Care Center, Anniston, USA

**Keywords:** central core disease, congenital myopathy, congenital neuromuscular disorder, malignant hyperthermia, rhabdomyolysis, ryr1 gene mutation, triple-negative breast cancer

## Abstract

Central core disease (CCD) is a congenital myopathy characterized by muscle weakness, skeletal deformities, delayed motor milestones, and susceptibility to malignant hyperthermia. Symptoms of CCD can be exacerbated by various physiological and pharmacological triggers, necessitating careful monitoring, particularly during medical interventions. Breast cancer treatment typically involves modalities such as surgery, radiation, chemotherapy, immunotherapy, and endocrine therapy, all of which may exacerbate underlying myopathic conditions. Chemotherapy and immunotherapy especially have the potential of causing myopathy, myositis, or rhabdomyolysis. Therefore, patients with CCD undergoing breast cancer treatment require vigilant surveillance to mitigate the risks associated with disease exacerbation. We report the case of a 41-year-old female patient with coexisting CCD and breast cancer who underwent neoadjuvant chemotherapy and immunotherapy, followed by surgical intervention and radiation therapy. Given her underlying myopathy, she was at an elevated risk for treatment-related complications, requiring regular monitoring of serum creatine kinase, myoglobin, and aldolase levels throughout her treatment course. The patient completed treatment without exacerbation of her myopathy and is currently in remission after two cycles of maintenance immunotherapy. For her surgery, surgeons were cautioned to take all necessary precautions to prevent malignant hyperthermia by avoiding certain anesthetics. This case highlights the importance of early intervention, close monitoring, and multidisciplinary management in patients with concurrent CCD and breast cancer.

## Introduction

Breast cancer remains one of the most common cancers among women in the United States, with approximately one in eight affected during their lifetime [1]. Among its various subtypes, triple-negative breast cancer (TNBC) is considered the most aggressive. Although relatively uncommon, representing about 10-15% of all breast cancer cases, TNBC is known for its rapid growth and high potential for metastasis. A 23% risk of recurrence and metastasis has been seen with TNBC, significantly higher than that seen with other subtypes of breast cancer [2]. Given its aggressive nature, it is important to understand TNBC, its clinical presentation, and treatment options [3].

TNBC differs from the other subtypes in that it lacks expression of the three key receptors commonly involved in breast cancer growth: estrogen, progesterone, and human epidermal growth factor receptor 2 (HER2) [3]. Due to the absence of these targets, TNBC is associated with a poor prognosis and more limited treatment options compared to other forms of breast cancer. Current therapeutic approaches often include neo-adjuvant chemotherapy and immunotherapy, followed by surgery and radiation [4]. However, in patients with pre-existing myopathy, such treatments may worsen symptoms such as muscle weakness and peripheral neuropathy or lead to anesthetic complications, further restricting available therapies.

Central core disease (CCD) is a rare congenital myopathy characterized by the presence of central cores on muscle biopsy [5]. It is most commonly associated with mutations in the skeletal muscle ryanodine receptor (RYR1) gene, which predisposes patients to malignant hyperthermia, a potentially fatal reaction triggered by certain anesthetic agents and muscle relaxants [5]. CCD can also present as varying degrees of muscle weakness, hypotonia, skeletal abnormalities, mild facial weakness, and respiratory issues causing increased risk of respiratory infections. Additionally, some chemotherapeutic agents contribute to the loss of body mass and skeletal muscle atrophy, leading to cachectic myopathy [6]. In rare cases, chemotherapy as well as immunotherapy can also induce rhabdomyolysis, posing further risk for individuals with CCD [6,7]. However, neoadjuvant pembrolizumab + chemotherapy followed by adjuvant pembrolizumab confers benefits in response and survival outcomes versus alternative neoadjuvant treatments for early-stage TNBC [8]. Hence, patients with CCD and early-stage TNBC who require cancer treatment need appropriate adjustments and frequent monitoring.

In this report, we present the unique case of a patient with TNBC and CCD, where the treatment approach required careful modification to accommodate her underlying neuromuscular condition.

## Case presentation

**Figure 1:**
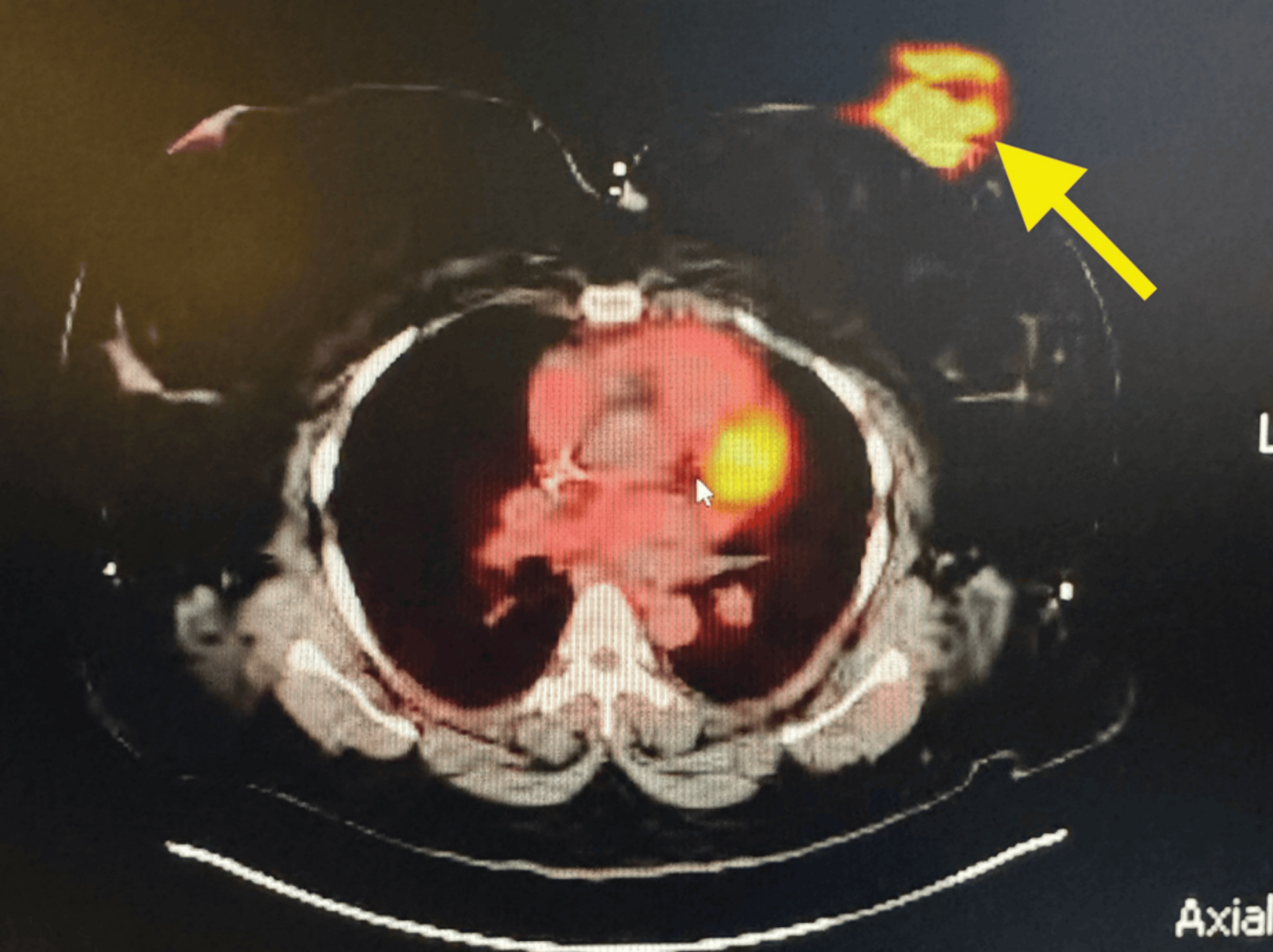
Axial PET/CT image demonstrating hyper-metabolic lesion in the left inner quadrant of the left breast (Slice 175/263) PET/CT: Positron Emission Tomography/Computed Tomography

**Figure 2:**
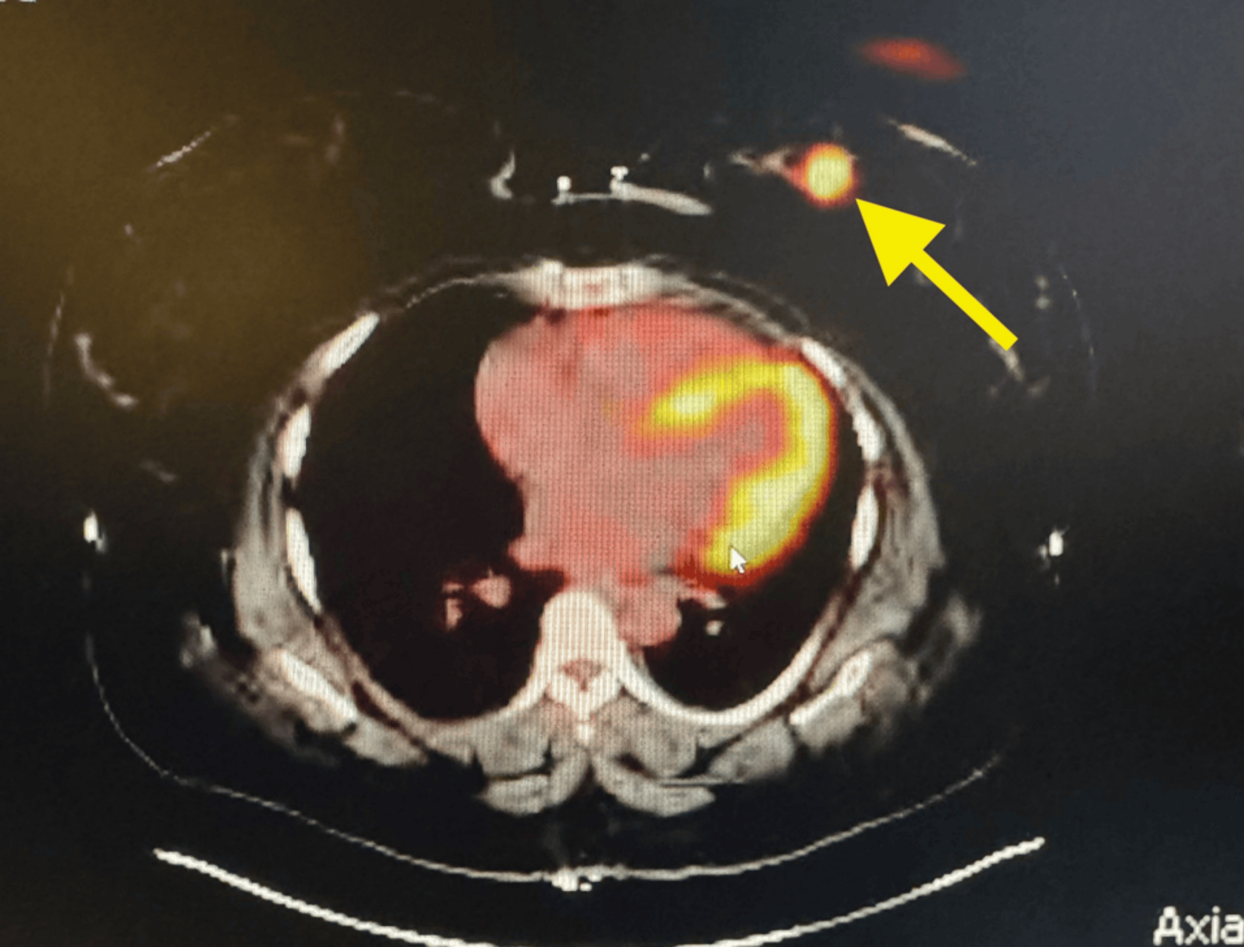
Axial PET/CT image demonstrating hyper-metabolic lesion in the left inner quadrant of the left breast (Slice 164/263) PET/CT: Positron Emission Tomography/Computed Tomography

A 41-year-old African-American female patient initially presented with a palpable left breast mass and associated discomfort. She was enrolled in the Alabama Breast and Cervical Cancer Early Detection Program (ABCCEDP), where a bilateral screening mammogram revealed a 2.5 cm mass in the lower inner quadrant (LIQ) of her left breast. A follow-up diagnostic mammogram and ultrasound demonstrated a 2.9 × 1.8 cm hypoechoic mass with microlobulated margins and associated left axillary lymphadenopathy, categorized as Breast Imaging Reporting and Data System Category 5 (BI-RADS 5). A core needle biopsy confirmed TNBC with estrogen receptor (ER) negative, progesterone receptor (PR) negative, and HER2 negative, which is histologically a poorly differentiated invasive ductal carcinoma. Her disease was staged as T2N1M0 based on imaging findings, including a Positron Emission Tomography/Computed Tomography (PET/CT) scan that showed intense 18F-fluorodeoxyglucose (FDG) uptake in the left breast and axillary nodes but no distant metastases, as can be seen in Figures 1, 2 (yellow arrows).

Prior to her presentation to our facility, the patient had undergone genetic testing at another clinic, which revealed a pathogenic RYR1 gene mutation consistent with CCD. Her daughter was also diagnosed with this genetic mutation and that prompted our patient's testing. Thus, her oncologic history was further complicated by this rare congenital neuromuscular disorder. The RYR1 gene mutation not only accounted for her lifelong muscle weakness and motor delays but also conferred a high risk of malignant hyperthermia in response to certain anesthetics. CCD has critical implications for perioperative care and chemotherapy-related myopathy risk. Additionally, she had a history of anemia, drug-induced neuropathy, and iron and folate deficiencies, requiring close hematologic monitoring throughout treatment. 

Given the complexity of managing TNBC in a patient with an underlying RYR1 gene mutation and CCD, a multidisciplinary treatment plan was implemented. Table 1 below summarizes the patient’s therapeutic course, including chemotherapy, immunotherapy, surgery, and radiation interventions, along with relevant supportive care.

**Table 1:** The different treatment modalities received by the patient during the course of disease management Supportive therapy was added to her regimen to manage her symptoms of central core disease.

Phase	Date(s)	Medications	Notes
Neoadjuvant (Keynote-522)	Feb-May 2024	Keytruda + Paclitaxel (Taxol) + Carboplatin	4 cycles: Keytruda q3w, Taxol/Carbo on D1, D8, D15
Post-neoadjuvant chemotherapy	May-July 2024	Epirubicin + Cyclophosphamide (Cytoxan) + Keytruda	4 cycles: Every 21 days, with Neulasta support
Supportive therapy	Ongoing	Iron, B12, Folic Acid, Gabapentin, Zofran, Imodium	To manage anemia, neuropathy, and chemo side effects
Radiation therapy	Jan-Apr 2025	External Beam Radiation	Completed; postmastectomy radiation
Maintenance immunotherapy	Ongoing through mid-2025	Keytruda q3w	9 total cycles post-surgery; 2 remaining as of April 2025
Surgery	Jan 2025	Bilateral mastectomy + sentinel node biopsy	Reconstruction planned

With confirmed TNBC and axillary lymph node involvement, treatment was initiated using the KEYNOTE-522 protocol, incorporating both chemotherapy and immunotherapy in the neoadjuvant setting. This included pembrolizumab, paclitaxel, and carboplatin, followed by pembrolizumab, cyclophosphamide, and epirubicin. The patient tolerated the regimen with expected side effects, including fatigue, neuropathy, anemia, and gastrointestinal symptoms. These were managed with supportive care, hydration, IV iron, folic acid, and vitamin B12 injections. Due to her severe anemia (Hb as low as 7.7 g/dL; normal 12-16 g/dL), she received transfusions and ongoing iron supplementation. Despite these complications, she demonstrated a significant clinical and radiographic response, and by August 2024, she reported no longer feeling the breast mass. 

To proactively manage the risk of muscle-related complications, particularly rhabdomyolysis and immune-mediated myositis, serial measurements of creatine kinase, myoglobin, and aldolase were obtained during therapy. A summary of these values is presented in Table 2 below.

**Table 2:** Monitoring creatine kinase (CK), myoglobin, and aldolase levels for early detection of any muscle related complications

Date	CK (U/L)			Myoglobin (ng/mL)			Aldolase (U/L)			Notes
	Pt value	Normal range	Threshold	Pt value	Normal range	Threshold	Pt value	Normal range	Threshold	
03/2024	105	22-198	>5,000 U/L	40	25-72	>300 ng/mL	3.5	1.0-7.5	>20-25 U/L	Baseline pre-therapy
05/2024	190	22-198	>5,000 U/L	69	25-72	>300 ng/mL	6.8	1.0-7.5	>20-25 U/L	Post-epirubicin + cytoxan
08/2024	175	22-198	>5,000 U/L	68	25-72	>300 ng/mL	5.2	1.0-7.5	>20-25 U/L	Post-chemo, pre-surgery
01/2025	193	22-198	>5,000 U/L	73	25-72	>300 ng/mL	7.3	1.0-7.5	>20-25 U/L	Post-surgery
04/2025	145	22-198	>5,000 U/L	55	25-72	>300 ng/mL	4.0	1.0-7.5	>20-25 U/L	End of radiation

All values remained within normal ranges throughout the course of treatment, with myoglobin going up to 73 once (normal range=25-72), but coming back to normal without any interventions.

She underwent bilateral mastectomy with sentinel node evaluation. The final pathology of the left breast revealed a 2.3 cm invasive ductal carcinoma with negative margins but tumor presence at the posterior inked margin; sentinel nodes were negative. The right breast showed no evidence of malignancy. Moreover, given her underlying susceptibility to malignant hyperthermia, dantrolene was made readily available prior to the administration of anesthetic agents as a precautionary measure. Postoperatively, she received radiation therapy, which she completed in April 2025, and was able to "ring the bell," marking the end of radiation. She has since resumed pembrolizumab maintenance every three weeks, with only two cycles remaining. Her post-treatment imaging, labs, and follow-up evaluations indicate continued remission, and she is scheduled to proceed with breast reconstruction in the coming months. This case emphasizes the importance of multidisciplinary coordination when managing cancer in patients with rare genetic disorders. It also highlights how personalized treatment plans can lead to excellent outcomes even in complex clinical scenarios.

## Discussion

Congenital myopathies affect approximately six out of every 100,000 live births, with CCD being one of the most common subtypes [9]. CCD is characterized by the presence of well-demarcated, oval-shaped cores within type 1 muscle fibers, which lack oxidative enzyme activity [9,10]. Clinically, it presents with features typical of congenital myopathy. The condition is associated with mutations in the RYR1 gene located on chromosome 19q13.1 [10].

The RYR1 gene encodes the ryanodine receptor, a calcium release channel within the sarcoplasmic reticulum (SR) of skeletal muscle. This channel regulates cytosolic calcium levels and plays a critical role in muscle excitation-contraction coupling. As seen in CCD, mutations in the RYR1 gene lead to clinical manifestations such as symmetrical mild weakness, hypotonia, delayed motor milestones, and predominant involvement of the proximal, axial, and hip girdle muscles. Facial weakness may also be present. Additionally, musculoskeletal deformities such as hip dislocation, scoliosis, and foot abnormalities are commonly observed [10].

Importantly, RYR1 mutations are also associated with a predisposition to malignant hyperthermia, with gain-of-function mutations found in 50-70% of affected individuals [11]. Malignant hyperthermia is a life-threatening hypermetabolic reaction of skeletal muscle triggered by exposure to certain volatile anesthetics or depolarizing muscle relaxants. It is characterized by uncontrolled calcium release from the SR, resulting in persistent muscle contraction, muscle rigidity, hyperthermia, hypercarbia, and cardiac arrhythmias [12].

The diagnosis of CCD typically involves a combination of physical examination, detailed family history, and muscle biopsy demonstrating central cores. Muscle MRI can reveal characteristic patterns of muscle involvement, especially when histopathology is inconclusive. Advances in genetic testing have allowed for the detection of RYR1 mutations, enabling a noninvasive and definitive diagnosis, and reducing the need for invasive procedures such as muscle biopsies [10].

In patients with CCD who develop breast cancer, management requires careful consideration of the potential side effects of chemotherapy, surgery, immunotherapy, and radiation therapy on underlying muscle pathology. Rhabdomyolysis, although rare, is a potentially fatal complication associated with certain chemotherapeutic agents. Previous reports have documented the development of rhabdomyolysis in patients after receiving chemotherapy [13,14]. Our patient was frequently monitored in order to prevent the development of any such complication. While the exact mechanism remains unclear, it is hypothesized that chemotherapeutic drugs may cause direct muscle membrane injury, leading to the release of intracellular enzymes such as creatine kinase and myoglobin [13]. Clinically, rhabdomyolysis can present with muscle pain, weakness, confusion, and, in severe cases, acute kidney injury. Agents such as paclitaxel, cytarabine, and cyclophosphamide have been implicated [14].

Similarly, immune checkpoint inhibitors, such as pembrolizumab, have been linked to rare cases of autoimmune myositis [15] and rhabdomyolysis [7]. Autoimmune myositis can present with muscle weakness (especially in the proximal muscles), muscle pain, and difficulty swallowing and breathing. Therefore, patients with CCD receiving these therapies require regular clinical assessment and monitoring of serum creatine kinase, myoglobin, and aldolase levels to detect early signs of muscle injury. Additionally, radiation-induced myopathy, although delayed, is another potential complication, often presenting years after therapy with muscle stiffness, weakness, and pain.

Surgical interventions, commonly necessary in breast cancer management, pose significant risks to patients predisposed to malignant hyperthermia. Volatile anesthetics such as halothane and isoflurane and depolarizing muscle relaxants such as succinylcholine are well-established triggers for malignant hyperthermia. The European Malignant Hyperthermia Group recommends the exclusive use of trigger-free anesthesia and ensuring that volatile anesthetic concentrations in the anesthesia workstation are kept at or below 5 parts per million (ppm) to prevent malignant hyperthermia in susceptible individuals [16].

This case highlights the unique challenges encountered when managing oncology patients with pre-existing myopathies. It emphasizes the critical role of early identification, individualized treatment planning, and vigilant monitoring for complications throughout cancer therapy. Through appropriate preventive strategies and close follow-up, the patient described in this report successfully completed her breast cancer treatment without exacerbation of her underlying myopathy, demonstrating the importance of proactive care in improving overall patient outcomes.

## Conclusions

This case highlights the complexities of managing breast cancer in a patient with underlying CCD, emphasizing the need for early recognition, multidisciplinary coordination, and careful monitoring. Despite being at high risk, she was able to receive appropriate chemotherapy and the KEYNOTE-522 protocol. Through individualized planning that included anesthetic precautions, regular surveillance of muscle biomarkers, and supportive management during chemotherapy and immunotherapy, the patient completed her treatment without significant myopathic complications. This report illustrates that, with appropriate preventive strategies, patients with rare genetic disorders can achieve successful oncologic outcomes while minimizing treatment-related risks.
